# Between Flesh and Identity: Embodied Selves in Disembodied Systems

**DOI:** 10.1007/s10508-025-03314-z

**Published:** 2025-09-24

**Authors:** Paolo Meneguzzo

**Affiliations:** 1 Department of Neuroscience, University of Padova, Via Giustiniani 2, 35128 Padua, Italy; 2 Padova Neuroscience Center, University of Padova, Padua, Italy

## Introduction

The questions “How many sexes are there?” and “How many genders are there?” seem, on the surface, deceptively simple. Yet in psychiatric and clinical work, they open a far-reaching inquiry—not just into biology and identity, but into how society constructs, regulates, and often invalidates human diversity. As a psychiatrist, working with transgender and gender-diverse (TGD) individuals and with those suffering from deep alienation from their own bodies—as seen in eating disorders, body dysmorphic disorders, and trauma—I encounter the limits and psychological suffering caused by binary categorization daily.

Psychiatry, especially in its social and developmental dimensions, is uniquely positioned to address these questions with both rigor and humility. Diagnostic frameworks, treatment approaches, and institutional practices are all influenced by prevailing understandings of sex and gender. When these frameworks reflect outdated or narrow models, they not only misclassify; they contribute to distress and impair psychological well-being.

From a psychiatric perspective, we must recognize that the denial of a person’s lived experience—of their gender, body, or identity—is not a neutral act. It is a form of symbolic violence, one that undermines self-coherence, increases psychological distress, and can lead to lasting psychiatric consequences. To reduce sex and gender to binaries is to erase lives that do not fit—in doing so, psychiatry fails its ethical mandate to care.

They kept asking me whether I was a boy or a girl. I said I was tired. I said I was 15. They didn’t hear me.

T., adolescent patient hospitalized after suicide attempt

## How Many Sexes Are There?

Biologically, sex has been framed as binary—male and female—based on chromosomal makeup and reproductive roles. Yet this view has long been contested by both science and medicine. Intersex individuals—those born with anatomical, chromosomal, or hormonal features that do not fit normative definitions of male or female—demonstrate that nature resists our neat categories. The reality of conditions such as 5-alpha-reductase deficiency, androgen insensitivity syndrome, Turner syndrome, and others underscores that sex is not a dimorphic variable, but a constellation of characteristics.

The binary model of sex erases the rich spectrum of human biological variation. Research across genetics, endocrinology, and developmental biology shows that definitions of sex can be based on multiple dimensions—chromosomal patterns, gonadal structure, hormonal profiles, and secondary sexual characteristics—which do not always align in a single individual (Ainsworth, 2015; Fausto-Sterling, 2000). Variations such as androgen insensitivity syndrome, congenital adrenal hyperplasia, and chromosomal differences like XXY or XO challenge the assumption that sex is reducible to a simple male–female dichotomy. From a clinical perspective, this complexity has concrete implications: It affects diagnostic practices, guides decisions in intersex medical care, and demands a move away from rigid categorization toward models that reflect biological diversity. Recognizing this variability not only improves scientific accuracy but also reduces the risk of harm from interventions aimed solely at enforcing binary norms.

Clinically, the implications are serious. The history of intersex medical care is fraught with nonconsensual surgeries and forced assignments designed to “normalize” bodies according to social expectations. These interventions often result in trauma, loss of sexual function, and psychological harm. Psychiatry’s role, historically complicit in these efforts, must now be one of restitution: to support rather than suppress embodied differences.

From a social-psychiatric perspective, sex is not only a matter of biology but a site of social meaning—of how bodies are interpreted, classified, and assigned value within cultural systems. Institutions such as the state, the clinic, and the family function as gatekeepers of this meaning, enforcing norms of legibility and punishing deviation. These structures dictate which bodies are seen as “coherent” or “natural,” and which are marked as anomalous or threatening. Those who do not conform to binary expectations risk becoming socially and culturally unintelligible: Their identities are positioned outside the dominant frameworks through which personhood is recognized (Butler, 2011). This marginalization operates not only at the symbolic level but also through structural mechanisms, resulting in barriers to healthcare, legal recognition, and the protection of fundamental rights. The denial of legibility thus functions both as a cultural exclusion and as a practical impediment to full participation in social life. Psychiatry, in this context, is not a neutral observer but a participant in this process of classification and exclusion—unless it consciously chooses otherwise.

## How Many Genders Are There?

Gender, in contrast to sex, is relational, developmental, and psychosocial. It is a way of being-in-the-world, structured through social norms but also experienced intimately. In this sense, asking “How many genders are there?” is akin to asking “How many ways are there to be a self?”—a question psychiatry must always answer with openness.

Gender identities develop within a complex web of relational and social influences that begin early in life and continue to evolve across the lifespan. Early attachment experiences, the narratives constructed within the family, peer relationships, and prevailing cultural scripts all contribute to shaping how individuals come to understand and express their gendered selves. Developmental perspectives have long recognized identity formation as a central psychosocial task, highlighting how supportive environments foster authenticity while unsupportive ones can lead to defensive or fragmented self-concepts. Processes such as mentalization play a critical role, enabling individuals to achieve a coherent sense of identity through the experience of being accurately perceived and understood by others. Within this framework, gender emerges not as a static designation imposed at birth, but as a dynamic, evolving dimension of selfhood, continuously negotiated in relation to others and to the broader sociocultural environment (Erikson, 1968; Fonagy et al., 2018; Winnicott, 2018).

Psychological, biological, and sociocultural evidence all point to the limitations of binary thinking. Converging research indicates that both sex and gender are better conceptualized as continua rather than fixed categories, a perspective that allows for the complexity and variability observed in human experience. For psychiatry, adopting this framework is not only a matter of conceptual accuracy but also a clinical necessity, as it fosters approaches that are more responsive to the diverse realities of patients’ lives (Hyde et al., 2019).

Importantly, gender diversity is not pathological. The DSM-5-TR explicitly clarifies that gender nonconformity is not in itself a mental disorder, but that distress may arise when identity is chronically invalidated or denied (American Psychiatric Association, 2022). From a clinical standpoint, the distinction is critical: What generates suffering in TGD individuals is not their identity, but the social responses to it. When families, institutions, and healthcare systems fail to affirm a person’s gender, they create environments of shame, fear, and disconnection. This is consistent with minority stress theory (Meyer, 2003), which demonstrates how systemic stigma leads to increased rates of anxiety, depression, suicidality, and substance use among TGD populations. These outcomes are not the result of inherent fragility or deviance, but of sustained exposure to environments that deny recognition. At the same time, other explanations have been proposed, including that gender dysphoria itself can be distressing because the incongruence between one’s experienced gender and physical body may remain a source of discomfort even in highly supportive social contexts (Schrager et al., 2024). These perspectives are not mutually exclusive and may interact in shaping the intensity and persistence of distress in different individuals.

When people use my name, my real name, it’s like I can breathe again. I’m still anxious, but at least I know I exist to them.

A., 28, nonbinary individual receiving outpatient care for generalized anxiety disorder

Evidence from multiple studies suggests that when TGD individuals receive affirmation and support, mental health and body image outcomes can improve. One large-scale investigation found that socially transitioned children displayed normative mental health scores, though the absence of pretransition data and the skew toward higher socioeconomic status limit causal conclusions (Olson et al., 2016). Access to gender-affirming interventions has been associated with reduced suicidality, underscoring the importance of timely care (Turban et al., 2020). More recent findings are less consistent: A two-year follow-up after puberty suppression showed no significant change in mental health (Olson-Kennedy et al., 2025), and a synthesis of existing research highlights that benefits vary across studies, depending on baseline psychological status, family support, and comorbidities (Karalexi et al., 2020; Miroshnychenko et al., 2025). Psychosocial context emerges as a particularly important factor; for example, longitudinal data show that internalized transphobia predicts a worse course of body uneasiness even in individuals undergoing gender-affirming hormone therapy (Castellini et al., 2023; Meneguzzo et al., 2024a). Across both research and clinical practice, the cumulative influence of relational environments—especially family oppositiveness, institutional discrimination, and community stigma—stands out as a critical determinant of well-being. Addressing these chronic stressors may be as vital to improving outcomes as any medical intervention.

## Psychiatry’s Role: Witnessing or Regulating?

Psychiatry has long been caught between two roles: that of witness and that of regulator. As a witness, it listens, contains, and affirms the complexity of human experience. As a regulator, it imposes norms, defines diagnostic categories, and controls access to care based on deviations from those norms. This tension is perhaps nowhere more acute than in the domain of sex and gender.

Historically, psychiatry, through its reliance on medical classification systems such as the DSM, has played a complicit role in the marginalization of gender-nonconforming individuals. Diagnostic categories such as gender identity disorder and transvestic fetishism reflected an era in which gender variance was pathologized, framing nonconformity as deviance or sexual perversion. These formulations mirrored broader societal anxieties about gender roles and the preservation of a rigid binary order, while positioning clinicians as gatekeepers of legitimacy. Access to care was often contingent on meeting diagnostic thresholds that reinforced normative expectations rather than responding to individual needs. The transition to gender dysphoria in the DSM-5-TR marked a shift in language and intent, but it still rests on a framework in which distress is the necessary criterion for recognition. This approach risks obscuring the fact that, for many, the primary source of suffering lies in external invalidation, systemic stigma, and structural barriers, not in their identity itself. As such, the classificatory lens of psychiatry continues to shape whose experiences are deemed real and worthy of care, sustaining a subtle but persistent asymmetry between gender-diverse individuals and the cisgender norm.

This framing risks positioning psychiatry as an institution that only sees what hurts, and remains blind to what affirms. It overlooks the many gender-diverse people who experience coherence, joy, and agency in their identities—individuals who do not need to be “cured,” but understood. Trans identity has been framed as a refusal to accept a world that seeks to impose predetermined roles and destinies, challenging the notion that it should be approached through a medicalized lens. This perspective underscores the importance for psychiatry of recognizing agency and self-determination as central to well-being rather than pathologizing them (Preciado, 2020). Social psychiatry urges us to move beyond the binary of pathologize or ignore. Instead, it calls for frameworks of structural competence (Metzl & Hansen, 2014)—approaches that recognize how history, power, and systemic bias shape what counts as disorder, and who is granted access to care and legitimacy.

## The Body as Social Text

The body, long before it becomes a psychiatric symptom, is a medium of communication—preverbal, affective, and relational. In both humans and other species, it signals safety, status, pain, and belonging.

In both eating disorders and gender dysphoria, I often encounter bodies that are not simply physical, but symbolic terrains: sites of negotiation between autonomy and control, recognition and shame, desire and rejection. Research on TGD populations shows that body objectification is not merely an esthetic concern but a mechanism through which social norms are enforced, intensifying body-related distress and mediating the link between external invalidation and internal suffering (Meneguzzo et al., 2024a, 2024b). The psychiatric symptom is not just internal; it is a response to social inscription.

If I can make my body disappear, maybe they’ll stop looking at it like it’s wrong.

F., 19, with anorexia nervosa and unrecognized gender dysphoria

When the body is denied—misnamed, pathologized, or erased—psychiatric symptoms often emerge. In this light, anorexia can be seen not only as a drive for thinness but as a desperate bid to gain control over a gendered, sexualized, and often unsafe body. Similarly, in gender-diverse individuals, distress frequently arises not from identity itself, but from the disruption between the felt self and the social gaze—a fracture psychiatry is uniquely placed to understand and repair.

Clinically, this underscores the need for an informed, socially aware psychiatry, one that understands identity as narrative and the body as a site of inscription. It also calls for humility: Psychiatry must be ready to question its own categories and ask whether they serve healing or merely reinforce norms.

## Conclusion

How many sexes are there? More than two—biologically, clinically, and ethically. How many genders? As many as there are ways to inhabit a body, a history, and a world. These questions cannot be answered solely through classification, because their meaning lies not in countability but in recognition. Biology reveals variation, while lived experience demands nuance. In psychiatry, our task is not to reduce people to categories, but to accompany them; not to diagnose difference, but to understand it; not to enforce conformity, but to foster meaning. Affirming gender and sex diversity is not a political gesture, but a clinical responsibility—and in doing so, psychiatry does not abandon scientific rigor; it reclaims its soul.

## Data Availability

Not applicable.
